# Supplementary material for: A Novel Three-Axial Magnetic-Piezoelectric MEMS AC Magnetic Field Sensor
Source: Micromachines (Basel). 2019 Oct 20;10(10):710. doi: 10.3390/mi10100710 (PMC6843634; doi:10.3390/mi10100710)
Supplement: Supplementary file 1 [file micromachines-10-00710-s001.pdf]

micromachines

Supplementary Materials

A Novel Three-Axial Magnetic-Piezoelectric MEMS AC Magnetic Field Sensor

Po-Chen Yeh [1], Hao Duan [1] and Tien-Kan Chung [1,2,*]

[1] Department of Mechanical Engineering, National Chiao Tung University, Hsinchu, 30010, Taiwan; pcyeh0203@gmail.com (P.-C.Y.); f19931031.me04g@nctu.edu.tw (H.D.)

[2] International College of Semiconductor Technology, National Chiao Tung University, Hsinchu, 30010, Taiwan;

* Correspondence: tkchung@nctu.edu.tw; Tel.: +886-03-5712121

The supplementary material includes:

Section I: Background Theories of Magnetic Force and Piezoelectric Voltage Output.
Section II: Frequency Response Measurement of the Sensor.
Section III: Test of Magnetic-Field Shielding Effect of the Magnetic-Field Shielding Box.
Figure S1. Resonant frequency testing setup using laser Doppler vibrometer.
Figure S2. The illustration and specification of Gauss meter and Gauss meter's probe used for magnetic-field measurement/calibration.
Figure S3. The testing setup and results of background magnetic-fields measurement.

Section I: Background Theories of Magnetic Force and Piezoelectric Voltage Output

Part I: Magnetic Force

Before we start deriving the governing equations of the magnetic force induced in the Ni thick film of our sensor, we need to mention that the governing equation capable of precisely calculating the attractive and repulsive magnetic forces between two magnets is very complex (it depends on the shape, magnetization, orientation, separation distance, and so on, of the magnets) and thus difficult to derive. Therefore, in order to avoid complex mathematic calculation/estimation, few assumptions are made to simplify the complex magnetic-force calculation/estimation for our sensor. These assumptions include: (1) The cylindrical Ni thick film on our sensor is considered as a cylindrical permanent magnet owning the magnetization-strength which is obtained from measurement. (2) The electromagnets used to produce the external AC magnetic field to the sensor are considered as cylindrical permanent magnets according to the Ampère model (note: in the Ampère model, the strength of a permanent magnet can be expressed in the same terms as that of an electromagnet and vice versa). Based on the above assumptions, we can simplify our model into a simple two-magnets system. To derive the approximated governing equation for our sensor, the Gilbert model [1] is used to express the magnetic force between the Ni thick film and the electromagnets. We chose the Gilbert model for magnetic force approximation for two reasons: First, the geometry of the Ni thick film and the electromagnet are both of cylindrical shape. Second, the distances between the Ni thick film and the electromagnets are much longer than their radii. These two reasons/conditions fit the basic assumptions of the Gilbert model for approximating the magnetic force between two cylindrical magnets. Therefore, the Gilbert model is appropriate to express the magnetic force in our case.

In the Gilbert model, the magnetic force between two cylindrical bar magnets, which are placed end-to-end at a very long distance ($x \gg R$, where x denotes the distance between two magnets and R denotes the radius of the magnet) with their magnetic dipole aligned, can be approximated as:

$$\mathrm{F}(x) \simeq \frac{\pi\mu_0}{4} M_1 M_2 {R_1}^2 {R_2}^2 \left[\frac{1}{x^2} + \frac{1}{(x+L_1 L_2)^2} - \frac{2}{\left(x+\frac{(L_1+L_2)}{2}\right)^2}\right] \tag{1}$$

where:

x is the distance between two magnets.

μ_0 is the permeability of space, which equals to $4\pi \times 10^{-7}$ T·m/A.

R_1 and R_2 are the radius of the Ni thick film and the electromagnets, respectively.

M_1 and M_2 are the magnetization of the Ni thick film and the electromagnets, respectively.

L_1 and L_2 are the length of the Ni thick film and the electromagnets, respectively.

According to Equation (1), the magnetic force between two cylindrical magnets (i.e., Ni thick film and electromagnets) is determined by their geometry with dimensions (i.e., R_1, R_2, L_1, L_2), distance between two magnets (i.e., x), and magnetizations (i.e., M_1 and M_2). However, because the geometry with the dimensions of the two magnets (i.e., R_1, R_2, L_1, L_2), the distance between two magnets (i.e., x), and the magnetization of the Ni thick film (i.e., M_1) are given/determined, the magnetic force between the sensor and the electromagnets is proportional to the electromagnets' magnetization (i.e., M_2; which varies with a magnitude-controllable current provided by a function generator). Therefore, by using the governing Equation (1), we can correlate the applied magnetic field generated from the electromagnets to the induced magnetic force exerted on the Ni thick film of the sensor. For a detailed derivation of Equation (1), please see [1]. In addition, we noticed that there are several different approximation equations reported by researchers to express the magnetic force between permanent magnets with different shapes (e.g., cuboidal and cylindrical) [2,3]. However, although these approximation equations might express the magnetic force as precisely as the Gilbert model, most of them are in a very complex form. Thus, the Gilbert model approximation is suitable for us to simply express the magnetic-field-induced magnetic force to our sensor.

Part II: Piezoelectric Voltage Output

After the magnetic force is induced in the Ni thick film, the relationship between the voltage and the magnetic force from the sensor must be estimated. To achieve this, the voltage output of a piezoelectric harvester/sensor can be approximated by using below governing Equation (2) (which is derived/reported by S. Roundy et al. and our previous work) [4,5]. Note that Equation (2), expressing the piezoelectric voltage output of a piezoelectric diaphragm, is estimated by a given driving vibration which is modified from Equation (45) in [5]. For a detailed derivation of Equation (2), please see [5].

$$V \simeq \frac{j\omega \frac{2c_p d_{31} t_c}{\varepsilon} \frac{A_{in}}{k}}{\left[\frac{\omega_n{}^2}{RC_b} - \left(\frac{1}{RC_b} + 2\zeta\omega_n\right)\omega^2\right] + \left[\omega_n{}^2\left(1 + k_{31}{}^2\right) + \frac{2\zeta\omega_n}{RC_b} - \omega_n{}^2\right]} \quad (2)$$

where:

ω is the frequency of driving vibration.

ω_n is the resonance frequency.

c_p is the elastic constant of the piezoelectric material.

d_{31} is the piezoelectric coefficient of the piezoelectric material.

k_{31} is the piezoelectric coupling coefficient of the piezoelectric material.

t_c is the thickness of the piezoelectric material.

k is a geometric constant relates strain to the deflection of the piezoelectric material.

ε is the dielectric constant of the piezoelectric material.

R is load resistance.

C_b is the capacitance of the piezoelectric material.

ζ is the unitless damping ratio.

A_{in} is the Laplace transform of the input vibrations in terms of acceleration.

The dielectric constant ε is calculated by using Equation (3) below [5]. The capacitance C_b is calculated by using below Equation (4) [5]. ζ is calculated from piezoelectric mechanical Q by using

the Equation (5). The geometric constant k is calculated from the relation of the stress and displacement of the diaphragm by using Equation (6) [5].

$$\varepsilon = \frac{{d_{31}}^2 c_p}{k_{31}} \tag{3}$$

$$C_b = \frac{\varepsilon {d_e}^2}{4t_c} \tag{4}$$

$$\zeta = \frac{1}{2Q} \tag{5}$$

$$k = \frac{16{t_c}^3}{\pi(1-\rho^2)} \tag{6}$$

where d_e is the diameter of the electrode covering the piezoelectric material (which equals to the diameter of the piezoelectric diaphragm in our case), t_c is the thickness of the piezoelectric diaphragm, and ρ is the Poisson's ratio of the piezoelectric material. According to the above Equations (2)–(6), since most parts of the parameters in Equation (2) are known by the given design and material properties of the sensor, the piezoelectric voltage output (V) is proportional to only one variable, the Laplace transform of the input vibration in terms of acceleration (A_{in}). In addition, according to Newton's second law of motion (i.e., $F = ma$), the acceleration of an object is proportional to the force acting on the object. Therefore, by using Equations (2)–(6), we can correlate the magnetic-field-induced force exerted on the Ni thick film of our sensor to the piezoelectric voltage output of our sensor. In addition, because of the proportional relation between the strength of the magnetic field and the induced magnetic force exerted on the sensor (as shown in Equation (1)) and the proportional relation between the magnetic force exerted on the sensor and the piezoelectric voltage output (as shown in Equation (2)–(6)), we can correlate them and conclude that there is also a proportional relation between the magnetic field to the piezoelectric voltage output. To clearly show this, the derivation process of the proportional relations of the above equations are shown below.

$$F \propto M_2 \tag{7}$$

$$F = ma \tag{8}$$

$$\mathrm{F} \propto a \tag{9}$$

$$V \propto A_{in} \tag{10}$$

$$V \propto M_2 \tag{11}$$

The derivation process starts from the magnetic force approximation Equation (1). In Equation (1), we can correlate the proportional relation between the induced magnetic force (i.e., F) exerted on the sensor and the strength of the magnetic field (i.e., M_2) applied to the sensor. The correlation is shown as Equation (7). After this, according to Newton's second law of motion as shown in Equation (8), the force (i.e., F) acting on the object is proportional to the acceleration (i.e., a) of an object. The proportional relation is shown as Equation (9). According to the piezoelectric voltage output approximation Equation (2), the piezoelectric voltage output (i.e., V) is proportional to the Laplace transform of the input vibration in terms of acceleration (i.e., A_{in}), as shown in Equation (10). Finally, according to the above derivation, the piezoelectric voltage output (i.e., V) is in proportional relation to the strength of the magnetic field (i.e. M_2) applied to the sensor, as shown in Equation (11). That is, Equation (11) represents the basic relation between the initial input (the magnetic field) and the final output (piezoelectric voltage) of our sensor. In addition, the proportional relation between the input and output of our sensor can explain the linearity of the magnetic field testing results of our sensor

as shown in Figure 9 in the manuscript. Furthermore, several articles regarding pressure oscillators/sensors using a piezoelectric diaphragm [6,7] proposed to show the proportional relation between the force exerted on the oscillator/sensor and the corresponding piezoelectric voltage output. In these articles, the experimental results show that the piezoelectric voltage output is linear to the pressure applied to the sensor. This confirms the proportional relation between the magnetic force and the final piezoelectric voltage output of our sensor.

Section II: Frequency Response Measurement of the Sensor

The frequency response of the fabricated mechanical diaphragm structure of a single sensing element was evaluated by using a laser Doppler vibrometer (LDV). The testing setup of the resonant frequency test is shown in Figure S1. Figure S1a shows the photograph of the LDV setup. Figure S1b illustrates the sensor mounted on the miniature piezoelectric stacked actuator. Figure S1c is the photograph of the miniature piezoelectric stacked actuator. The testing procedures are described as follows. First, we used double-side tapes to attach the fabricated single sensing element onto a miniature piezoelectric actuator, which is a standard testing device used to provide a vibrational frequency sweeping to a MEMS device during the resonant frequency test. After this, the laser spot of the LDV was aligned onto the center of the Ni thick film (which is also the center of the fabricated diaphragm) of the sensing element. After the alignment, a frequency sweep ranging from 1 Hz to 30 kHz was conducted by the miniature piezoelectric actuator. During the frequency sweeping, the frequency response of the sensing element is recorded by the LDV. This completes the resonant frequency test of our sensor.

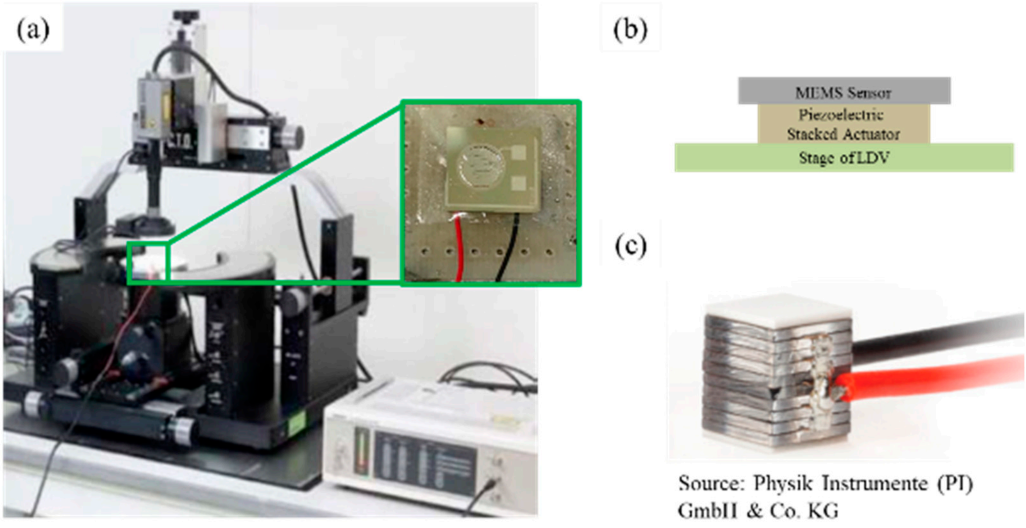

Figure S1. Resonant frequency testing setup using laser Doppler vibrometer (LDV): (**a**) photograph of the LDV setup, (**b**) illustration of the sensor mounted on the miniature piezoelectric stacked actuator for testing, and (**c**) photograph of the miniature piezoelectric stacked actuator.

Section III: Test of Magnetic Field Shielding Effect of the Magnetic Field Shielding Box

To see the actual effect contributed by the shielding box, we conduct a simple test to measure the strength of the background magnetic field inside and outside the shielding box. To measure the magnitude of the background magnetic fields (includes DC and AC magnetic fields), a Gauss meter (model: 5170, manufacturer: F. W. Bell Milwauki, OR, USA) and a Gauss meter's probe (model: STD18-0404, manufacturer: F. W. Bell, Milwauki, OR, USA) were used. The resolution and frequency bandwidth of the Gauss meter as 0.1 Gauss and 20 kHz, respectively. The specifications of the Gauss meter and Gauss meter's probe used for measurement are shown in Figure S2. The testing procedure is described as follows. First, we placed the Gauss meter probe inside the shielding box to measure the AC and DC background magnetic fields in three-axial directions. After this, we conducted the same measurements but outside the box. After the test was completed, we recorded and analyzed

the values of these background magnetic fields to figure out the magnetic field shielding effect of the shielding box. The testing setup and results of the background magnetic field measurement are shown in Figure S3a–b and Figure S3c, respectively. As shown in Figure S3c, the testing results show that the DC background magnetic fields in X-axial, Y-axial, and Z-axial inside the shielding box were 0.3 ± 0.1 Gauss, 0.2 ± 0.1 Gauss, and 0.3 ± 0.1 Gauss, respectively. In contrast, the DC background magnetic fields in X-axial, Y-axial, and Z-axial outside the shielding box were 0.5 ± 0.1 Gauss, 0.5 ± 0.1 Gauss, and 0.3 ± 0.1 Gauss, respectively. As for the AC background magnetic fields, all the measurement results indicate that there were no AC background magnetic fields inside or outside the shielding box (or the AC background magnetic fields were too small to be detected). Therefore, due to the above testing results, we can conclude that the magnetic shielding box is able to shield the DC magnetic fields by a magnitude of 0.1 to 0.2 Gauss. Nevertheless, these small magnitudes of DC magnetic fields (shielded or unshielded) do not influence the sensing results of our sensor. This is because the targeted sensing magnetic fields of our sensor are AC magnetic fields. In addition, because we utilized the lock-in amplifier to achieve the phase-oriented signal amplification, this small DC bias from the background magnetic fields becomes negligible. Therefore, based on the above reasons, our sensor (without shielding box) can sense three-axial magnetic fields as well as other magnetic sensors. This means that our sensor (without shielding box) can be used for most actual applications as well as other magnetic sensors. (Note: this claim is valid when the sensor is away from sources of strong magnetic-field interferences such as high-power electrical instruments or permanent magnets). Nevertheless, when conducting the laboratory-level tests, we think that it is good to have the shielding box to provide a standard near-zero magnetic field testing environment (free of magnetic field noises) for the sensor, as a standard testing reference.

(a)

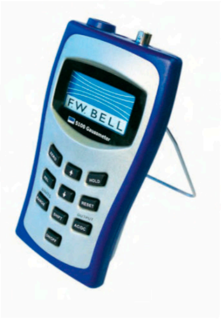

5100 Series **Specifications**

Model	5170	5180
Basic DC Accuracy	2%	1.1%
Frequency Bandwidth	DC-20 kHz	DC-25 kHz
**Update Rates**		
Display	4 readings/sec	4 readings/sec
Analog Output	None	100K samples/sec
**Ranges**		
Ultra Low Range (low-field probe only)	1 G	1 G
Low Range	200 G	300 G
Mid Range	2 kG	3 kG
High Range	20 kG	30 kG
**Resolution**		
Ultra Low Range (low-field probe only)	1 mG	1 mG
Low Range	0.1 G	0.1 G
Mid Range	1.0 G	1.0 G
High Range	10 G	10 G

(b)

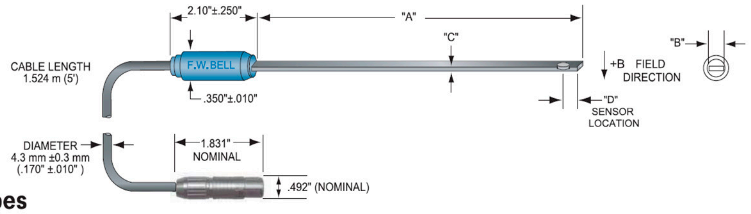

Transverse Probes

Model	A ±.063"	B	C	D	Material	Corrected Linearity	Sensitivity	Active area	Operating temp. range	Temp. stability (typical) Zero	Temp. stability (typical) Calibrate	Frequency Response
STD18-0402	2"	0.158" ±.004"	0.045" ±.004"	0.0335" (NOMINAL)	Polypro-pylene **see note	0.5%/30kG	1X	0.015" DIA. (NOM)	0°C to +75°C	±0.300 Gauss / °C (typical)	-0.05% % / °C (typical)	DC to 20kHz
STD18-0404	4"											
STH17-0402	2"					1.0%/20kG						DC to 10kHz
STH17-0404	4"											
HTH17-0604	4"	0.180" ±.004"	0.060" +.000" -.004"		ALUMINUM 3003 3/4 FH	1.0%/20kG						DC to 10kHz
HTD18-0604	4"					0.5%/30kG						DC to 20kHz
STB1X-0201	1"	.050" ±.005"	.020" MAX	0.030" (NOMINAL)	KAPTON	1.0%/10kG		0.025" DIA (NOM.)		±0.200 Gauss / °C	–0.1 % / °C	DC only

Note: Due to continuous process improvement, specifications subject to change without notice.
** Prior to late 2006 Transverse Probe Stems were rigid glass epoxy, .150 x .040".

Figure S2. The illustration and specification of (**a**) Gauss meter and (**b**) Gauss meter's probe used for magnetic field measurement/calibration.

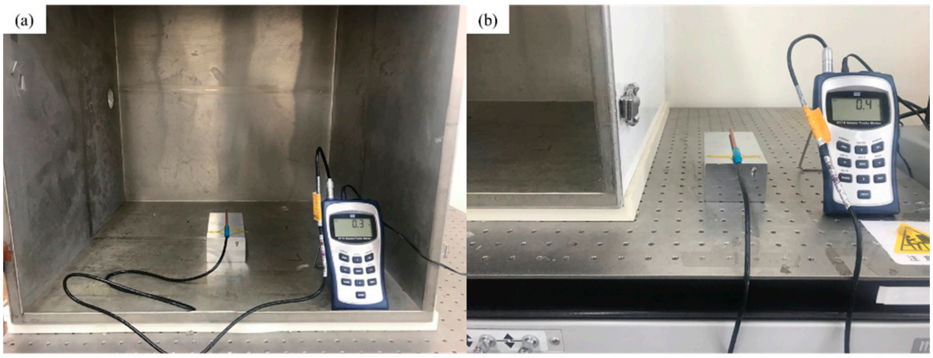

(c)

	Inside The Shielding Box		Outside The Shielding Box	
	DC	AC	DC	AC
X-axial (Gauss)	0.3 ± 0.1	0 ± 0.0	0.5 ± 0.1	0 ± 0.0
Y-axial (Gauss)	0.2 ± 0.1	0 ± 0.0	0.5 ± 0.1	0 ± 0.0
Z-axial (Gauss)	0.3 ± 0.1	0 ± 0.0	0.3 ± 0.1	0 ± 0.0

Figure S3. The testing setup and results of background magnetic-fields measurement. Photographs of testing setup (**a**) inside the shielding box and (**b**) outside the shielding box. (**c**) Table summarizing the testing results.

References

1. Vokoun, D.; Beleggia, M.; Heller, L.; Sittner, P. Magnetostatic interactions and forces between cylindrical permanent magnets. *J. Magn. Magn. Mater.* **2009**, *321*, 3758–3763.
2. Ravaud, R.; Lemarquand, D.; Babic, S.; Lemarquand, V.; Akyel, C. Cylindrical magnets and coils: Fields, forces, and inductances. *IEEE T. Magn.* **2010**, *46*, 3585–3590.
3. Agashe, J.S.; Arnold, D.P. A study of scaling and geometry effects on the forces between cuboidal and cylindrical magnets using analytical force solutions. *J. Phys. D Appl. Phys.* **2008**, *41*, 105001.
4. Hung, C.F.; Chung, T.K.; Yeh, P.C.; Chen, C.C.; Wang, C.M.; Lin, S.H. A miniature mechanical-piezoelectric-configured three-axis vibrational energy harvester. *IEEE Sens. J.* **2015**, *15*, 5601–5615, doi:10.1109/JSEN.2015.2444993.
5. Roundy, S.; Wright, P.K. A piezoelectric vibration based generator for wireless electronics. *Smart Mater. Struct.* **2004**, *13*, 1131–1142.
6. Ishiguro, Y.; Zhu, J.; Okubo, T.; Tagawa, N.; Okubo, K. Piezoelectric characteristic analysis of diaphragm type PZT oscillator. In Proceedings of the 2016 IEEE International Ultrasonics Symposium Proceedings, Tours, France, 18–21 September 2016.
7. Deshpande, M.; Saggere, L. An analytical model and working equations for static deflections of a circular multi-layered diaphragm-type piezoelectric actuator. *Sens. Actuators A* **2007**, *136*, 673–689.

© 2019 by the authors. Submitted for possible open access publication under the terms and conditions of the Creative Commons Attribution (CC BY) license (http://creativecommons.org/licenses/by/4.0/).
